# Energy Metabolism-Related Gene Prognostic Index Predicts Biochemical Recurrence for Patients With Prostate Cancer Undergoing Radical Prostatectomy

**DOI:** 10.3389/fimmu.2022.839362

**Published:** 2022-02-24

**Authors:** Dechao Feng, Xu Shi, Facai Zhang, Qiao Xiong, Qiang Wei, Lu Yang

**Affiliations:** Department of Urology, Institute of Urology, West China Hospital, Sichuan University, Chengdu, China

**Keywords:** energy metabolism, prostate cancer, tumor immune microenvironment, biochemical recurrence, immune checkpoint

## Abstract

**Background:**

We aimed to construct and validate an energy metabolism-related gene prognostic index (EMRGPI) to predict biochemical recurrence (BCR) in patients undergoing radical prostatectomy.

**Methods:**

We used Lasso and COX regression analysis to orchestrate the EMRGPI in the TCGA database, and the prognostic value of EMRGPI was further validated externally using the GSE46602. All analyses were conducted with R version 3.6.3 and its suitable packages.

**Results:**

SDC1 and ADH1B were finally used to construct the risk formula. We classified the 430 tumor patients in the TCGA database into two groups, and patients in the high-risk group had a higher risk of BCR than those in the low-risk group (HR: 1.98, 95%CI: 1.18-3.32, p=0.01). Moreover, in the GSE46602, we confirmed that the BCR risk in the high-risk group was 3.86 times higher than that in the low-risk group (95%CI: 1.61-9.24, p=0.001). We found that patients in the high-risk group had significantly higher proportions of residual tumor, older age, and T stage. SDC1 and ADH1B were significantly expressed low in the normal tissues when compared to the tumor tissues, which were opposite at the protein level. The spearman analysis showed that EMRGPI was significantly associated with B cells, CD4+ T cells, CD8+ T cells, neutrophils, macrophages, dendritic cells, stromal score, immune score, and estimate score. In addition, the EMRGPI was positively associated with the 54 immune checkpoints, among which CD80, ADORA2A, CD160, and TNFRSF25 were significantly related to the BCR-free survival of PCa patients undergoing RP.

**Conclusions:**

The EMRGPI established in this study might serve as an independent risk factor for PCa patients undergoing radical prostatectomy.

## Introduction

With the population aging, the overall health burden of prostate cancer (PCa) is increasing. Radical prostatectomy (RP) remains the first choice for the treatment of localized PCa. However, nearly 50% of patients encounter biochemical recurrence (BCR) after surgery (1). The current definition of BCR is heterogeneous, the most predictive threshold for metastasis after RP is PSA>0.4 ng/ml (2). After the radical radiotherapy, regardless of short-term hormone control, the definition of BCR is any PSA increase >2 ng/ml higher than the PSA nadir, regardless of the nadir value (3). It is believed that the impact of BCR on survival is only limited to a subgroup of patients with specific clinical risk factors (4). However, the prognosis of patients with BCR varies. Thus, indications for further treatment should not be based solely on meeting the threshold defined above for PSA, but rather a prediction method of individualized progression risk of PCa patients (5).

The occurrence of BCR is based on multiple systematic pathway alterations. In the process of tumor transformation, prostate cells undergo metabolic reprogramming to meet the needs of growth and proliferation. Metabolomics provides a down-stream measurement. Lucarelli et al. summarized that the PCa metabolome was characterized by accumulation of metabolic intermediates and increased expression of genes in the Krebs cycle, induction of *de novo* lipogenesis and cholesterol production (6, 7). Clendinen et al. proposed a nomogram to predict BCR through metabolomics, and found that many pathways altered, including amino acid metabolism, purine and pyrimidine synthesis, tricarboxylic acid (TCA) cycle, tryptophan catabolism, glucose, and lactate, and the lipid abundance was higher among BCR patients for a number of classes, including triglycerides, lysophosphatidylcholines, phosphatidylethanolamines, phosphatidylinositols, diglycerides, acyl carnitines, and ceramides (8). Studying the metabolic changes of the prostate is helpful to distinguish the indolent tumors from aggressive tumors, and to predict BCR.

Previous studies have reported several gene biomarker models to predict BCR for PCa patients undergoing RP (9–13), but the large number of genes in the model limits their clinical application. Adequate energy metabolism is essential for the survival of tumor cells. For the first time, we constructed and validated an energy metabolism-related gene prognostic index (EMRGPI) using only two genes to predict BCR in PCa patients undergoing RP. Our study has been registered in the ISRCTN registry (No. ISRCTN11560295).

## Methods

### Data Preparation

We downloaded and integrated PCa data from the UCSC XENA and the previous study (14, 15). We extracted the matrix of message RNA (mRNA) and identified the tumor-related genes through weighted gene co-expression network analysis (WGCNA). The significantly relevance was defined as lcoefficientl > 0.3 and p < 0.05. Differentially expressed genes (DEGs) were analyzed, which were considered as llogFCl > 1 and padj < 0.01. Two energy metabolism-related gene sets (energy-requiring part of metabolism and reactome energy metabolism) were obtained from the molecular signature database (MsigDB, http://www.broad.mit.edu/gsea/msigdb/) (16). Subsequently, the candidate genes were identified through the intersection of tumor-related genes, DEGs and energy metabolism-related genes. We used the Lasso and COX regression analysis to figure out the independent risk genes associated with BCR-free survival, and then orchestrated the energy metabolism-related gene prognostic index (EMRGPI). The EMRGPI risk score= 0.348*SDC1+0.229*ADH1B. Patient data undergoing RP in the GSE46602 (17) were downloaded from the Gene Expression Omnibus (GEO) (18), and were further used to externally confirm the prognostic value of EMRGPI. In addition, we confirmed the differential expression of SDC1 and ADH1B at protein level through the human protein atlas (HPA) database (19, 20).

### Function Analysis and Tumor Immune Environment (TME) Analysis

The genes interacted with SDC1 and ADH1B was analyzed through the GeneMANIA database (21). We divided the 430 tumor patients into high- and low-risk group according to the median of EMRGPI. Gene set enrichment analysis (GSEA) was conducted to explore the possible pathways (16, 22). Considering gene expression profile and risk groups, the minimum gene set was 5 and maximum was 5000. P < 0.05 and false discovery rate (FDR) < 0.10 were considered statistically significant.

We used the TIMER and ESTIMATE algorithms (23, 24) to analyze the TME of PCa patients. The spearman analysis was used to analyze the correlations between EMRGPI and TME parameters and 54 common immune checkpoints. We also explored the prognostic values of the checkpoints related to the EMRGPI in predicting BCR-free survival.

### Statistical Analysis

We performed all analyses using software R 3.6.3 and its suitable packages. We utilized Wilcoxon test under the circumstance of non-normal data distribution. Variables could be entered into multivariate COX regression analysis if p value < 0.1 in the univariable Cox regression analysis. Survival analysis was conducted through log-rank test and presented as Kaplan-Meier curve. Besides, the Spearman analysis was used to assess the correlations among continuous variables if they did not meet Shapiro-Wilk normality test. Statistical significance was set as two-sided p < 0.05. Significant marks were as follows: no significance (ns), p≥0.05; *, p< 0.05; **, p<0.01; ***, p<0.001.

## Results

### EMRGPI and Its Clinical Values

**Figure 1:**
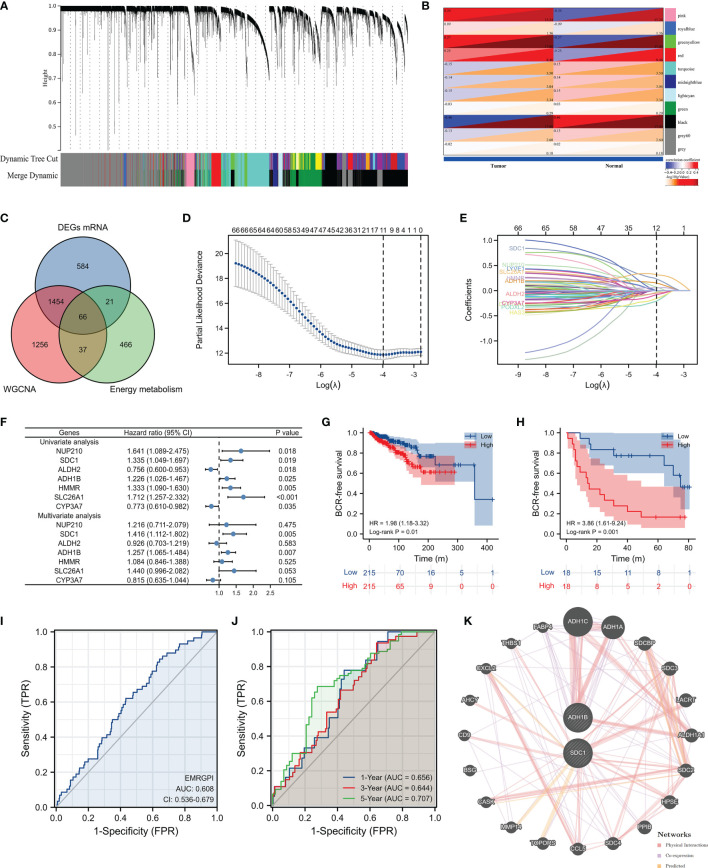
Identification of EMRGPI and its clinical values. **(A)** gene cluster plot showing the process of WGCNA analysis; **(B)** modules and phenotype showing 2183 genes in the black, greenyellow, and pink modules which were highly related to tumor; **(C)** Venn plot showing the intersection of tumor-related genes, DEGs and energy metabolism-related genes; **(D)** variables screening through the Lasso regression analysis where the lambda value was 0.0185; **(E)** trajectory diagram of the 11 genes identified through the Lasso regression analysis; **(F)** forest plot showing the COX regress analysis of genes associated with BCR-free survival; **(G)** Kaplan-Meier curve showing survival difference of high- and low-risk group in the TCGA database; **(H)** Kaplan-Meier curve showing survival difference of high- and low-risk group in the GSE46602 (17); **(I)** ROC curve showing the diagnostic ability of EMRGPI in distinguishing BCR from no BCR; **(J)** Time-dependent ROC curve showing the diagnostic ability of EMRGPI in distinguishing BCR from no BCR; **(K)** Gene interacted with ADH1B and SDC1. BCR, biochemical recurrence; DEGs, differentially expressed genes; EMRGPI, energy metabolism-related gene prognostic index; WGCNA, weighted gene co-expression network analysis; mRNA, message RNA; ROC, receiver operating characteristic curve. prostate cancer patients were divided into high- and low-risk groups according to the median of the EMRGPI score.

We obtained 498 tumor and 52 normal samples of PCa from the TCGA database, among which 430 PCa patients undergoing RP had complete data of BCR (**Supplementary Table 1**). Patients who experienced BCR were significantly associated with higher Gleason score and advanced T stages (**Supplementary Table 1**). We clustered the genetic mRNA expression of 498 tumor and 52 normal samples of PCa from the TCGA database using the WGCNA analysis (**Figure 1A**), and identified 2183 genes in the black, greenyellow, and pink modules which were highly related to tumor (**Figure 1B**). 66 candidate genes were found through the intersection of tumor-related genes, DEGs and energy metabolism-related genes (**Figure 1C**). 11 genes were found through the Lasso regression analysis using the methods of 10-fold cross-validation, where the lambda value was 0.0185 (**Figure 1D**). We also presented the trajectory diagram of the 11 genes in **Figure 1E**. A total of 7 of the 11 genes were significantly associated with BCR-free survival, and multivariate COX regression analysis was conducted using the 7 genes (**Figure 1F**). SDC1 and ADH1B were the independent risk factors of PCa patients, and we further constructed the risk formula using the two genes. We classified the 430 tumor patients in the TCGA database into two groups according to the median of the EMRGPI score, and patients in the high-risk group had a higher risk of BCR than those in the low-risk group (HR: 1.98, 95%CI: 1.18-3.32, p=0.01; **Figure 1G**). We further observed that the EMRGPI could serve as the independent risk factor of BCR for PCa patients through the multivariate COX regression analysis which enrolled the EMRGPI and clinical indicators in the TCGA database (**Supplementary Table 2**). Moreover, PCa patients in the GSE46602 (17) were divided into high- and low-risk groups based on the median of EMRGPI score, and we confirmed that the BCR risk in the high-risk group was 3.86 times higher than that in the low-risk group (95%CI: 1.61-9.24, p=0.001; **Figure 1H**). The diagnostic ability of EMRGPI distinguishing BCR patients from no BCR patients in the TCGA database was low (**Figures 1I, J**). Physical interactions and co-expression between ADH1B, and ADH1C, ADH1A and ALDH2 were observed, and CXCL2, MMP14, and TOPORS were predicted to interacted with SDC1 (**Figure 1K**). The age of high-risk group was significantly higher than that of low-risk group (61.58 ± 6.61 vs 60.29 ± 6.86, p=0.047; **Table 1**). Moreover, we found that patients in the high-risk group had significantly higher proportions of residual tumor (p=0.016), and T stage (p < 0.001) (**Table 1**).

**Table 1:** The correlations between EMGPI and clinical parameters in the TCGA database.

Characteristic	Low-risk group	High-risk group	P value
Sample (n)	215	215	
Age, mean ± SD	60.29 ± 6.86	61.58 ± 6.61	0.047
BCR, n (%)			0.034
No	194 (45.1%)	178 (41.4%)	
Yes	21 (4.9%)	37 (8.6%)	
N stage, n (%)			0.310
N0	152 (40.5%)	154 (41.1%)	
N1	29 (7.7%)	40 (10.7%)	
Positive lymphnodes, n (%)			0.249
No	144 (40.2%)	144 (40.2%)	
Yes	29 (8.1%)	41 (11.5%)	
Residual tumor, n (%)			0.016
No	151 (36%)	122 (29.1%)	
Yes	62 (14.8%)	84 (20%)	
Gleason score, n (%)			0.066
GS=6	23 (5.3%)	16 (3.7%)	
GS=7	113 (26.3%)	93 (21.6%)	
GS=8	26 (6%)	33 (7.7%)	
GS=9	53 (12.3%)	73 (17%)	
T stage, n (%)			<0.001
T2	98 (23.1%)	57 (13.4%)	
T3-4	115 (27.1%)	154 (36.3%)	
Race, n (%)			0.088
ASIAN	9 (2.2%)	2 (0.5%)	
Black or African American	26 (6.2%)	24 (5.8%)	
White	172 (41.3%)	183 (44%)	

EMRGPI, energy metabolism-related gene prognostic index; BCR, biochemical recurrence; GS, Gleason score; SD, standard deviation.

### Differential Expression of SDC1 and ADH1B and TME Analysis

**Figure 2:**
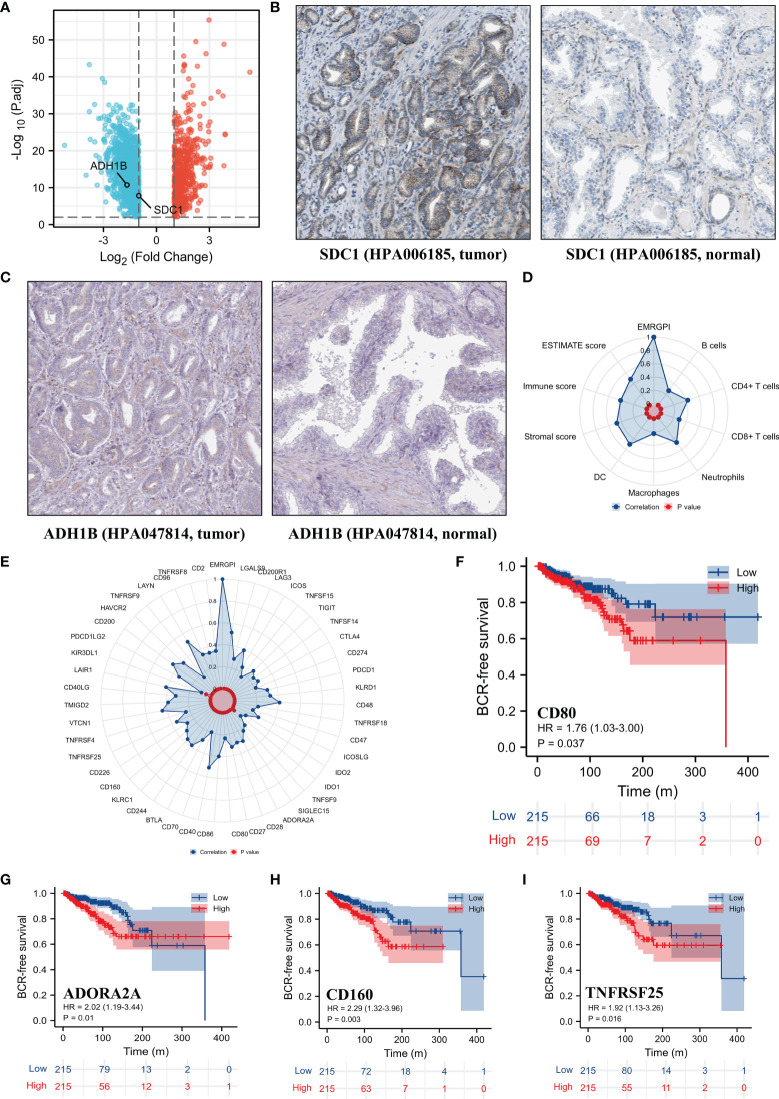
Differential expression of SDC1 and ADH1B and TME analysis. **(A)** volcano plot showing differentially expressed genes between tumor and normal tissues; **(B)** differential expression of SDC1 at protein level in the HPA database (19, 20); **(C)** differential expression of ADH1B at protein level in the HPA database (19, 20); **(D)** the correlations between EMRGPI and TME indicators; **(E)** the correlations between EMRGPI and immune checkpoints; **(F)** Kaplan-Meier curve showing survival difference of high- and low-expression of CD80 in the TCGA database; **(G)** Kaplan-Meier curve showing survival difference of high- and low-expression of ADORA2A in the TCGA database; **(H)** Kaplan-Meier curve showing survival difference of high- and low-expression of CD160 in the TCGA database; **(I)** Kaplan-Meier curve showing survival difference of high- and low-expression of TNFRSF25 in the TCGA database. BCR, biochemical recurrence; EMRGPI, energy metabolism-related gene prognostic index; TME, tumor immune microenvironment.

The mRNA expression of SDC1 and ADH1B were significantly lower in the tumor tissues when compared to the normal tissues (**Figure 2A**), which were opposite at the protein levels through the HPA database (19, 20) (**Figures 2B, C**). The spearman analysis showed that EMRGPI was significantly associated with B cells (r: 0.27), CD4+ T cells (r: 0.42), CD8+ T cells (r: 0.29), neutrophils (r: 0.47), macrophages (r: 0.22), dendritic cells (r: 0.55), stromal score (r: 0.47), immune score (r: 0.41), and estimate score (r: 0.48) (**Figure 2D**). In addition, the EMRGPI was positively associated with the 54 immune checkpoints (**Figure 2E**), among which CD80 (HR: 1.76, 95%CI: 1.03-3.00, p=0.037; **Figure 2F**), ADORA2A (HR: 2.02, 95%CI: 1.09-3.44, p=0.01; **Figure 2G**), CD160 (HR: 2.29, 95%CI: 1.32-3.96, p=0.003; **Figure 2H**), and TNFRSF25 (HR: 1.92, 95%CI: 1.13-3.26, p=0.016; **Figure 2I**) were significantly related to the BCR-free survival of PCa patients undergoing RP.

### Functional Enrichment Analysis

430 PCa patients in the TCGA database were classified into two groups according to the median of the EMRGPI score, and the results of GSEA analysis between low- and high-risk group were presented in **Table 2**. Several cancers, such as thyroid cancer, renal cell carcinoma, and small lung cancer, were enriched in high-risk group. In terms of signaling pathways, insulin, chemokine, WNT, T cell receptor, MAPK, and NOD like receptor signaling pathways were highly upregulated in high-risk group. In addition, several cellular and molecular processes, including regulation of actin cytoskeleton, snare interactions in vesicular transport, apoptosis, focal adhesion, extracellular matrix (ECM) receptor interaction, FC gamma R-mediated phagocytosis, endocytosis, and cell adhesion molecules, were enriched in the high-risk group.

**Table 2:** The results of gene set enrichment analysis between low- and high-risk group.

Gene set enrichment analysis (low vs high)	ES	NES	P value	FDR
**Diseases**				
Thyroid cancer	-0.5641	-1.7859	0.002	0.0993
Renal cell carcinoma	-0.4976	-1.7256	0.0061	0.0857
Chronic myeloid leukemia	-0.4632	-1.9046	0.004	0.0913
Small cell lung cancer	-0.5052	-1.6768	0.002	0.0902
Viral myocarditis	-0.6277	-1.6275	0.0179	0.0951
Prion diseases	-0.6151	-1.6357	0.0096	0.0948
Amyotrophic lateral sclerosis	-0.6664	-2.0416	0	0.0667
**Signaling pathways**				
Insulin signaling pathway	-0.422	-1.6726	0.0041	0.0859
Chemokine signaling pathway	-0.6329	-1.674	0.002	0.0887
WNT signaling pathway	-0.5035	-1.6844	0.002	0.0945
T cell receptor signaling pathway	-0.6221	-1.6316	0.0222	0.0954
MAPK signaling pathway	-0.4928	-1.6858	0	0.099
NOD like receptor signaling pathway	-0.6707	-1.6584	0.0118	0.0889
Epithelial cell signaling in helicobacter pylori infection	-0.4971	-1.7791	0.0095	0.0928
**Cellular and molecular processes**				
Aminoacyl tRNA biosynthesis	0.671	2.0732	0.002	0.0069
Protein export	0.6717	1.9508	0.004	0.0136
Terpenoid backbone biosynthesis	0.7399	1.9039	0.0043	0.0159
ECM receptor interaction	-0.67	-1.7404	0	0.084
Cell adhesion molecules	-0.6756	-1.6688	0	0.0845
Glycerophospholipid metabolism	-0.4809	-1.6577	0.0036	0.0861
Apoptosis	-0.5184	-1.7541	0	0.0862
FC gama R-mediated phagocytosis	-0.5799	-1.7315	0.006	0.0867
Focal adhesion	-0.5713	-1.7456	0.002	0.0875
Endocytosis	-0.4043	-1.7156	0.008	0.0891
Leukocyte transendothelial migration	-0.5816	-1.6832	0	0.0904
Snare interactions in vesicular transport	-0.4596	-1.7564	0.0094	0.0917
Regulation of actin cytoskeleton	-0.5287	-1.7681	0	0.0922
Vascular smooth muscle contraction	-0.5444	-1.6403	0.0039	0.0946

ECM, extracellular matrix; ES, enrichment score; NES, Normalized enrichment score; FDR, false discovery rate.

## Discussion

Although surgery or radiotherapy can effectively improve the prognosis of PCa patients and prolong their survival, the rate of recurrence and metastasis remains high. Meanwhile, there may be a tendency of over-medical treatment for the large population of PCa patients (25). Magnetic resonance imaging variables, prostate-specific antigen (PSA), and Gleason score are currently common mainstream methods for predicting BCR (26, 27). It is currently recommended that PSA doubling time and pathological Gleason score are indicators used to grade the risk of BCR after RP (5). Actually, we observed that BCR patients had higher Gleason score and advanced T stages than no BCR patients in this study. Maxeiner et al. used magnetic-resonance-spectroscopy-based metabolomic profiles to establish a model for predicting BCR through changes in several metabolites including spermine/polyamines, glutamine, myo-inositol, phosphoryl choline, scylloinositol, and glutamate, with an accuracy of 78% (28). Stabler et al. used the combination of serum PSA with cystathionine, cysteine, and homocysteine as markers to predict BCR with an AUC of 0.86 (29). In this paper, from the perspective of energy metabolism, we firstly found individual approach of gene-level recurrence markers that are helpful to the clinical decision-making of PCa patients. Furthermore, compared to the previous gene signatures (9–13), we included two different genes in our study and provided a simpler prognostic gene formula from the perspective of energy metabolism.

Like other metabolic cancers, increased glycolysis can provide more metabolic intermediates and energy for the rapid proliferation of PCa cells (30). Shao et al. observed significant accumulation of metabolic intermediates in PCa and the enrichment of genes in the TCA cycle, indicating that the TCA cycle in PCa tissue is over-activated, and existence of potential replenishment pathways for the metabolism of pyruvate, glutamine and branched chain amino acids in PCa supplements the metabolites of the TCA cycle (31). Androgen receptor (AR) plays an important role in increasing glycolysis in PCa cells, which can induce flux through the classical TCA cycle and reductive carboxylation of glutamine (32–34). The AR constitutively actives splice variants, such as AR-V7, stimulates glycolysis to a similar degree to AR in changing metabolism, and at the same time improves the utilization of citrate, and possibly metabolize it into other compounds needed for cell growth, such as lipids, steroids and amino acids, which increases the tumor’s ability to grow (34). It has been shown that androgens can stimulate AMPK-PGC1α cascade by increasing mitochondrial function and biogenesis, and activate glycolysis and oxidative phosphorylation (OXPHOS) (33). Clendinen et al. found that lactate and other end products of glucose catabolism increased in patients with BCR (1). The increased lactate may be related to the Warburg effect (35). Meanwhile, tumor cells induce the secretion of lactate and pyruvate by cancer-associated fibroblasts (CAFs) through aerobic glycolysis, and then they take up these energy-rich metabolites to promote efficient energy production through mitochondrial OXPHOS, thereby producing higher proliferation capacity, the reverse Warburg effect (36). This process of lactate exchange between CAFs and cancer cells is called lactate shuttle (37). In addition, different literatures also reported the relationship between elevated methionine metabolites such as cysteine and BCR (8, 29).

Alcohol dehydrogenase family (ADH1B and ADH1C) metabolize a wide variety of substrates, including ethanol, retinol, other aliphatic alcohols, hydroxysteroids, and lipid peroxidation products (38). ADH1B (rs1229984) and aldehyde dehydrogenase 2 (ALDH2) (rs671) are the two main genes involved in ethanol metabolism (39, 40). Genetic polymorphisms of ADH1B, ADH1C and ALDH2 have been reported involving in the development and progression of many cancers, such as gastric cancer (41), head and neck cancers (42), esophageal cancer (43), and pancreatic cancer (44). In a mendelian randomization study, it was found that in ALDH1B1 (rs10973794) was associated with PCa mortality with low-grade prostate cancer (HR = 1.43; p = 0.002) (45). So far, epidemiologic evidence for association between alcohol intake and the risk of PCa still remain unclear. Many articles, meta-analyses and systematic reviews showed contradictory conclusions (46–49). The possible reason might be the gene polymorphism which was associated with the enzyme activity. SDC1 was found to be significantly associated with BCR for PCa patients undergoing RP (50, 51), which could mutually confirm with our results. Moreover, serum SDC-1 levels have also been confirmed to be related to PCa progression, overall survival, disease specific survival, and chemotherapy resistance (52, 53). The inflammation of tumor patients is not limited to the local tumor, but systemic inflammation, clinically manifested as increased myeloid cells, and neutrophil-to-lymphocyte ratio in the circulation is closely related to poor prognosis in cancers (54). Systemic mobilization of neutrophils promotes metastatic diffusion, while SDC1 shedding is a critical endogenous mechanism that facilitates the resolution of neutrophilic inflammation by aiding the clearance of proinflammatory chemokines (like CXCL12) in a heparan sulfate-dependent manner (54, 55). MMP14 is up-regulated in PCa cells, and may be involved in mediating the mutual crosstalk between PCa cells and periprostatic adipose tissue, promoting tumor invasion (56). SDC1 could inhibit early stages of liver fibrogenesis by interfering with TGFβ1 action and upregulating MMP14 (57). Besides, TOPORS is a ubiquitously expressed E3 ubiquitin ligase that can ubiquitinate the tumor suppressor gene p53 (58). Notably, we found that the transcriptional and protein levels of these two genes were completely opposite in this study, which indicated the role of epigenetic or post-transcriptional regulation.

The overexpression of focal adhesion kinase is associated with the formation and invasive activity of androgen-independent PCa cells (59). The activation of FAK/src/paxillin/Rac/JNK leads to an increase in the activity of matrix metalloproteinases and the reorganization of membrane molecules, changes in adhesion to collagen type I and invasion into collagen type I, and may be one of the mechanisms of PCa invasion (60). The remodeling of collagen ECM is thought to be related to aging and PCa growth and invasion, since the collagen matrix extracted from aged mice enhances the invasion and proliferation of PCa cells *in vitro* (61). Reactive stroma where metabolites and genes linked to immune functions and ECM remodeling are significantly upregulated is a common tissue feature in the TME of PCa and are also associated with BCR (62). The MAPK signaling pathway can be triggered by growth factors such as TGF-β, leading to the down-regulation of epithelial markers and the up-regulation of mesenchymal markers, resulting in epithelial-mesenchymal transition (EMT) (63–65). The activation of the non-canonical Wnt pathway induced by Wnt5a/Fzd2 is significantly related to EMT and metastasis, and has been proven to be an important predictor of BCR (66). This feature is also related to the decreased concentration of metabolites citrate and spermine, which are thought to be associated with aggressive PCa (66). A nomogram constructed based on the Wnt ligand gene family is used to predict BCR, and the C index is 0.719 (67). In addition, we also found that EMRGPI was related to chronic myelogenous leukemia, thyroid cancer, renal cell carcinoma, and small cell lung cancer through functional analysis, further proving its clinical relevance.

In this study, we observed that EMGPI was positively associated with the immune infiltrating cells and TME scores. We thought that the metabolic competition between cancer cells and immune cells inhibited the function of immune cells and the metabolic reprogramming also played a significant role in suppressing the immune attack on the tumor cells and in resistance to therapies (68). Lactate is an immunosuppressive molecule, whose elevation in PCa cells and TME could promote the immune escape (69). Meanwhile, lactate inhibits the differentiation of monocytes and dendritic cells, and induces the inactivation of cytotoxic T lymphocytes (35). Moreover, the elevated lactate in TME can promote the polarization of tumor-related macrophages (TAMs) to M2 by activating the ERK/STAT3 signaling pathway (70), and tumor cells tend to survive and metastasize through its secretion of anti-inflammatory and promoting angiogenesis cytokines (71). Excessive production of pro-inflammatory cytokines and extracellular matrix-related molecules leads to the lipolysis of cancer cells to produce free fatty acids, which induce oxidative stress through the expression of pro-oxidant enzyme NADPH oxidase 5 (56). Then, increased reactive oxygen species production activates the HIF1/MMP14 pathway, which contributes to the invasion ability of PCa cells (56). At the same time, we observed that EMRGPI was correlated with stromal score. The interaction between tumor and stroma is also believed to play a role in the metabolic reprogramming of tumor cells. It is worth noting that this ability to induce metabolic reprogramming is bidirectional. CAF is induced to up-regulate the expression of the glucose transporter GLUT1, enhance the production and the output of lactate through the *de novo* expression of monocarboxylic acid transporter 4 (72). At the same time, after PCa cells are in contact with CAF, the expression of GLUT1 decreases, and the input of lactate through the lactate transporter MCT1 increases and then lactate enters the TCA cycle (72). The so-called reverse Warburg effect describes a metabolic symbiosis model in which CAF provides energy and metabolites for epithelial cancer cells (72). In tumor stroma, matrix components. including CAFs establish a metabolic symbiosis relationship with PCa cells through lactate shuttle and cellular bridges both *in vitro* and *in vivo*, which ultimately leads to a high exploitation of mitochondria, TCA cycle deregulation and enhanced PCa invasiveness (73). Other stromal components such as adipocytes are also believed to possess a similar metabolic symbiosis relationship and are believed to be related to PCa metastasis (74, 75). In TRAMP+/p62adipo mice, obesity and more aggressive PCa are shown. At the same time, energy expenditure pathways such as lipogenesis and OXPHOS in adipose tissue are inhibited to save energy substrates for FA β-oxidation gene-enriched PCa cells, with an up-regulated level of the rate-limiting enzyme of the transport of long-chain FAs for β-oxidation, CPT1A, thus promoting EMT and cancer aggressiveness (76).

We also found positive correlations between EMRGPI and many checkpoints, among which CD80, ADORA2A, CD160, and TNFRSF25 were highly associated with BCR-free survival. Adenosine mediates immune suppression in the TME by ADORA2A on immune cells. Drugs targeting ADORA2A have entered phase I clinical trials for the immunotherapy of patients with renal cell carcinoma (77). Serum CD80 is related to BCR (78). CD160 is essential for NK-mediated IFN-γ production (79). For hepatocellular carcinoma, the reduction in the number and function of CD160 + NK cells in TME contributes to the immune escape (80). Members of the TNF receptor superfamily (TNFRSF) are the key co-stimulators of T cells, and TNFRSF25 can promote CD8⁺ T cell responses and anti-tumor immunity (81).

For the first time, our article proposed genes related to energy metabolism to predict BCR of PCa patients undergoing RP. It not only provided the latest insights to the correlations between cancer cells and TME cells, but most importantly, it proposed a method for screening high-risk BCR patients at the genetic level, which was helpful for individualized screening of early treatment patient groups, and ultimately helped to reduce PCa medical costs. However, the potential mechanism of the opposite difference between transcriptional and protein levels is needed to be further studied. Besides, the role of energy metabolism between tumor cells and immune cells still warranted to be investigated.

## Conclusions

The EMRGPI established in this study might serve as an independent risk factor for PCa patients undergoing RP.

## Data Availability Statement

The datasets presented in this study can be found in online repositories. The names of the repository/repositories and accession number(s) can be found in the article/**Supplementary Material**.

## Author Contributions

DF proposed the project, conducted data analysis, interpreted the data, and wrote the manuscript. XS, FZ, QX, and QW, conducted data analysis, interpreted the data. LY supervised the project, and interpreted the data. All authors reviewed and edited the manuscript. All authors contributed to the article and approved the submitted version.

## Funding

This program was supported by the National Natural Science Foundation of China (Grant Nos. 81974099, 82170785, 81974098, 82170784), programs from Science and Technology Department of Sichuan Province (Grant Nos. 21GJHZ0246), Young Investigator Award of Sichuan University 2017 (Grant No. 2017SCU04A17), Technology Innovation Research and Development Project of Chengdu Science and Technology Bureau (2019-YF05-00296-SN), Sichuan University–Panzhihua science and technology cooperation special fund (2020CDPZH-4). The funders had no role in study design, data collection or analysis, preparation of the manuscript, or the decision to publish.

## Conflict of Interest

The authors declare that the research was conducted in the absence of any commercial or financial relationships that could be construed as a potential conflict of interest.

## Publisher’s Note

All claims expressed in this article are solely those of the authors and do not necessarily represent those of their affiliated organizations, or those of the publisher, the editors and the reviewers. Any product that may be evaluated in this article, or claim that may be made by its manufacturer, is not guaranteed or endorsed by the publisher.

## Supplementary Material

Supplementary Table 1 The baselines of patients with prostate cancer from the TCGA database. BCR, biochemical recurrence; GS, Gleason score; IQR, interquartile range.

Supplementary Table 2 Univariate and multivariate COX regression analysis of EMGPI and clinical indicators for the paitents with prostate cancer from the TCGA database. EMRGPI, energy metabolism-related gene prognositc index; GS, Gleason score.

## Abbreviations

EMRGPI, energy metabolism-related gene prognostic index; RP, radical prostatectomy; PCa, prostate cancer; BCR, biochemical recurrence; TCA, tricarboxylic acid; PSA, prostate-specific antigen; OXPHOS, oxidative phosphorylation; CAF, cancer-associated fibroblast; EMT, epithelial-mesenchymal transition; TME, tumor immune microenvironment; TAM, tumor-associated macrophage; ECM, extracellular matrix; AR, androgen receptor; WGCNA, weighted gene co-expression network analysis; DEGs, differentially expressed genes; mRNA, message RNA; GSEA, gene set enrichment analysis.

## References

[1] Suardi N Porter CR Reuther AM Walz J Kodama K Gibbons RP. A Nomogram Predicting Long-Term Biochemical Recurrence After Radical Prostatectomy. Cancer (2008) 112(6):1254–63. doi: 10.1002/cncr.23293 18286530

[2] Amling CL Bergstralh EJ Blute ML Slezak JM Zincke H. Defining Prostate Specific Antigen Progression After Radical Prostatectomy: What Is the Most Appropriate Cut Point? J Urol (2001) 165(4):1146–51. doi: 10.1016/S0022-5347(05)66452-X 11257657

[3] Roach M 3rd Hanks G Thames H Jr Schellhammer P Shipley WU Sokol GH. Defining Biochemical Failure Following Radiotherapy With or Without Hormonal Therapy in Men With Clinically Localized Prostate Cancer: Recommendations of the RTOG-ASTRO Phoenix Consensus Conference. Int J Radiat Oncol Biol Phys (2006) 65(4):965–74. doi: 10.1016/j.ijrobp.2006.04.029 16798415

[4] Van den Broeck T van den Bergh RCN Arfi N Gross T Moris L Briers E. Prognostic Value of Biochemical Recurrence Following Treatment With Curative Intent for Prostate Cancer: A Systematic Review. Eur Urol (2019) 75(6):967–87. doi: 10.1016/j.eururo.2018.10.011 30342843

[5] Van den Broeck T van den Bergh RCN Briers E Cornford P Cumberbatch M Tilki D. Biochemical Recurrence in Prostate Cancer: The European Association of Urology Prostate Cancer Guidelines Panel Recommendations. Eur Urol Focus (2020) 6(2):231–4. doi: 10.1016/j.euf.2019.06.004 31248850

[6] Lucarelli G Rutigliano M Galleggiante V Giglio A Palazzo S Ferro M. Metabolomic Profiling for the Identification of Novel Diagnostic Markers in Prostate Cancer. Expert Rev Mol Diagn (2015) 15(9):1211–24. doi: 10.1586/14737159.2015.1069711 26174441

[7] Lucarelli G Loizzo D Ferro M Rutigliano M Vartolomei MD Cantiello F. Metabolomic Profiling for the Identification of Novel Diagnostic Markers and Therapeutic Targets in Prostate Cancer: An Update. Expert Rev Mol Diagn (2019) 19(5):377–87. doi: 10.1080/14737159.2019.1604223 30957583

[8] Clendinen CS Gaul DA Monge ME Arnold RS Edison AS Petros JA. Preoperative Metabolic Signatures of Prostate Cancer Recurrence Following Radical Prostatectomy. J Proteome Res (2019) 18(3):1316–27. doi: 10.1021/acs.jproteome.8b00926 30758971

[9] Luan J Zhang Q Song L Wang Y Ji C Cong R. Identification and Validation of a Six Immune-Related Gene Signature for Prediction of Biochemical Recurrence in Localized Prostate Cancer Following Radical Prostatectomy. Transl Androl Urol (2021) 10(3):1018–29. doi: 10.21037/tau-20-1231 PMC8039594 33850736

[10] Zhang L Li Y Wang X Ping Y Wang D Cao Y. Five-Gene Signature Associating With Gleason Score Serve as Novel Biomarkers for Identifying Early Recurring Events and Contributing to Early Diagnosis for Prostate Adenocarcinoma. J Cancer (2021) 12(12):3626–47. doi: 10.7150/jca.52170 PMC8120165 33995639

[11] Shao N Tang H Mi Y Zhu Y Wan F Ye D. A Novel Gene Signature to Predict Immune Infiltration and Outcome in Patients With Prostate Cancer. Oncoimmunology (2020) 9(1):1762473. doi: 10.1080/2162402X.2020.1762473 32923125 PMC7458664

[12] Long X Hou H Wang X Liu S Diao T Lai S. Immune Signature Driven by ADT-Induced Immune Microenvironment Remodeling in Prostate Cancer Is Correlated with Recurrence-Free Survival and Immune Infiltration. Cell Death Dis (2020) 11(9):779. doi: 10.1038/s41419-020-02973-1 32951005 PMC7502080

[13] Luan JC Zhang QJ Zhao K Zhou X Yao LY Zhang TT. A Novel Set of Immune-Associated Gene Signature Predicts Biochemical Recurrence in Localized Prostate Cancer Patients After Radical Prostatectomy. J Cancer (2021) 12(12):3715–25. doi: 10.7150/jca.51059 PMC8120173 33995646

[14] Goldman MJ Craft B Hastie M Repečka K McDade F Kamath A. Visualizing and Interpreting Cancer Genomics Data *via* the Xena Platform. Nat Biotechnol (2020) 38(6):675–8. doi: 10.1038/s41587-020-0546-8 PMC7386072 32444850

[15] Liu J Lichtenberg T Hoadley KA Poisson LM Lazar AJ Cherniack AD. An Integrated TCGA Pan-Cancer Clinical Data Resource to Drive High-Quality Survival Outcome Analytics. Cell (2018) 173(2):400–16.e11. doi: 10.1016/j.cell.2018.02.052 29625055 PMC6066282

[16] Subramanian A Tamayo P Mootha VK Mukherjee S Ebert BL Gillette MA. Gene Set Enrichment Analysis: A Knowledge-Based Approach for Interpreting Genome-Wide Expression Profiles. PNAS (2005) 102(43):15545–50. doi: 10.1073/pnas.0506580102 PMC1239896 16199517

[17] Mortensen MM Høyer S Lynnerup AS Ørntoft TF Sørensen KD Borre M. Expression Profiling of Prostate Cancer Tissue Delineates Genes Associated With Recurrence After Prostatectomy. Sci Rep (2015) 5:16018. doi: 10.1038/srep16018 26522007 PMC4629186

[18] Edgar R Domrachev M Lash AE. Gene Expression Omnibus: NCBI Gene Expression and Hybridization Array Data Repository. Nucleic Acids Res (2002) 30(1):207–10. doi: 10.1093/nar/30.1.207 PMC99122 11752295

[19] Uhlen M Zhang C Lee S Sjöstedt E Fagerberg L Bidkhori G. A Pathology Atlas of the Human Cancer Transcriptome. Science (2017) 357(6352):eaan2507. doi: 10.1126/science.aan2507 28818916

[20] Uhlén M Fagerberg L Hallström BM Lindskog C Oksvold P Mardinoglu A. Proteomics. Tissue-Based Map of the Human Proteome. Science (2015) 347(6220):1260419. doi: 10.1126/science.1260419 25613900

[21] Warde-Farley D Donaldson SL Comes O Zuberi K Badrawi R Chao P. The GeneMANIA Prediction Server: Biological Network Integration for Gene Prioritization and Predicting Gene Function. Nucleic Acids Res (2010) 38(Web Server issue):W214–20. doi: 10.1093/nar/gkq537 PMC2896186 20576703

[22] Liberzon A Subramanian A Pinchback R Thorvaldsdóttir H Tamayo P Mesirov JP. Molecular Signatures Database (MSigDB) 3.0. Bioinformatics (2011) 27(12):1739–40. doi: 10.1093/bioinformatics/btr260 PMC3106198 21546393

[23] Li B Severson E Pignon JC Zhao H Li T Novak J. Comprehensive Analyses of Tumor Immunity: Implications for Cancer Immunotherapy. Genome Biol (2016) 17(1):174. doi: 10.1186/s13059-016-1028-7 27549193 PMC4993001

[24] Yoshihara K Shahmoradgoli M Martínez E Vegesna R Kim H Torres-Garcia W. Inferring Tumour Purity and Stromal and Immune Cell Admixture From Expression Data. Nat Commun (2013) 4:2612. doi: 10.1038/ncomms3612 24113773 PMC3826632

[25] Etzioni R Penson DF Legler JM di Tommaso D Boer R Gann PH. Overdiagnosis Due to Prostate-Specific Antigen Screening: Lessons From U.S. Prostate Cancer Incidence Trends. J Natl Cancer Inst (2002) 94(13):981–90. doi: 10.1093/jnci/94.13.981 12096083

[26] Hu XH Cammann H Meyer HA Jung K Lu HB Leva N. Risk Prediction Models for Biochemical Recurrence After Radical Prostatectomy Using Prostate-Specific Antigen and Gleason Score. Asian J Androl (2014) 16(6):897–901. doi: 10.4103/1008-682X.129940 25130472 PMC4236336

[27] Poulakis V Witzsch U de Vries R Emmerlich V Meves M Altmannsberger HM. Preoperative Neural Network Using Combined Magnetic Resonance Imaging Variables, Prostate-Specific Antigen, and Gleason Score for Predicting Prostate Cancer Biochemical Recurrence After Radical Prostatectomy. Urology (2004) 64(6):1165–70. doi: 10.1016/j.urology.2004.06.030 15596191

[28] Maxeiner A Adkins CB Zhang Y Taupitz M Halpern EF McDougal WS. Retrospective Analysis of Prostate Cancer Recurrence Potential With Tissue Metabolomic Profiles. Prostate (2010) 70(7):710–7. doi: 10.1002/pros.21103 PMC2909586 20017167

[29] Stabler S Koyama T Zhao Z Martinez-Ferrer M Allen RH Luka Z. Serum Methionine Metabolites Are Risk Factors for Metastatic Prostate Cancer Progression. PloS One (2011) 6(8):e22486. doi: 10.1371/journal.pone.0022486 21853037 PMC3154200

[30] Vander Heiden MG Cantley LC Thompson CB. Understanding the Warburg Effect: The Metabolic Requirements of Cell Proliferation. Science (2009) 324(5930):1029–33. doi: 10.1126/science.1160809 PMC2849637 19460998

[31] Shao Y Ye G Ren S Piao HL Zhao X Lu X. Metabolomics and Transcriptomics Profiles Reveal the Dysregulation of the Tricarboxylic Acid Cycle and Related Mechanisms in Prostate Cancer. Int J Cancer (2018) 143(2):396–407. doi: 10.1002/ijc.31313 29441565

[32] Massie CE Lynch A Ramos-Montoya A Boren J Stark R Fazli L. The Androgen Receptor Fuels Prostate Cancer by Regulating Central Metabolism and Biosynthesis. EMBO J (2011) 30(13):2719–33. doi: 10.1038/emboj.2011.158 PMC3155295 21602788

[33] Tennakoon JB Shi Y Han JJ Tsouko E White MA Burns AR. Androgens Regulate Prostate Cancer Cell Growth *via* an AMPK-PGC-1alpha-Mediated Metabolic Switch. Oncogene (2014) 33(45):5251–61. doi: 10.1038/onc.2013.463 PMC4009392 24186207

[34] Shafi AA Putluri V Arnold JM Tsouko E Maity S Roberts JM. Differential Regulation of Metabolic Pathways by Androgen Receptor (AR) and Its Constitutively Active Splice Variant, AR-V7, in Prostate Cancer Cells. Oncotarget (2015) 6(31):31997–2012. doi: 10.18632/oncotarget.5585 PMC4741655 26378018

[35] Nenu I Gafencu GA Popescu T Kacso G. Lactate - A New Frontier in the Immunology and Therapy of Prostate Cancer. J Cancer Res Ther (2017) 13(3):406–11. doi: 10.4103/0973-1482.163692 28862200

[36] Pavlides S Whitaker-Menezes D Castello-Cros R Flomenberg N Witkiewicz AK Frank PG. The Reverse Warburg Effect: Aerobic Glycolysis in Cancer Associated Fibroblasts and the Tumor Stroma. Cell Cycle (2009) 8(23):3984–4001. doi: 10.4161/cc.8.23.10238 19923890

[37] Brooks GA. Lactate: Glycolytic End Product and Oxidative Substrate During Sustained Exercise in Mammals — The “Lactate Shuttle. In: Gilles R, editor. Circulation, Respiration, and Metabolism. Berlin Heidelberg: Springer Nature. (1985). p. 208–18.

[38] Stelzer G Rosen N Plaschkes I Zimmerman S Twik M Fishilevich S. The GeneCards Suite: From Gene Data Mining to Disease Genome Sequence Analyses. Curr Protoc Bioinf (2016) 54:1.30.1–1.30.33. doi: 10.1002/cpbi.5 27322403

[39] Seitz HK Stickel F. Molecular Mechanisms of Alcohol-Mediated Carcinogenesis. Nat Rev Cancer (2007) 7(8):599–612. doi: 10.1038/nrc2191 17646865

[40] Crabb DW Edenberg HJ Bosron WF Li TK. Genotypes for Aldehyde Dehydrogenase Deficiency and Alcohol Sensitivity. The Inactive ALDH2(2) Allele Is Dominant. J Clin Invest (1989) 83(1):314–6. doi: 10.1172/JCI113875 PMC303676 2562960

[41] Hidaka A Sasazuki S Matsuo K Ito H Sawada N Shimazu T. Genetic Polymorphisms of ADH1B, ADH1C and ALDH2, Alcohol Consumption, and the Risk of Gastric Cancer: The Japan Public Health Center-Based Prospective Study. Carcinogenesis (2015) 36(2):223–31. doi: 10.1093/carcin/bgu244 25524923

[42] Chang JS Straif K Guha N. The Role of Alcohol Dehydrogenase Genes in Head and Neck Cancers: A Systematic Review and Meta-Analysis of ADH1B and ADH1C. Mutagenesis (2012) 27(3):275–86. doi: 10.1093/mutage/ger073 22042713

[43] Wu M Chang SC Kampman E Yang J Wang XS Gu XP. Single Nucleotide Polymorphisms of ADH1B, ADH1C and ALDH2 Genes and Esophageal Cancer: A Population-Based Case-Control Study in China. Int J Cancer (2013) 132(8):1868–77. doi: 10.1002/ijc.27803 PMC4122263 22930414

[44] Mohelnikova-Duchonova B Vrana D Holcatova I Ryska M Smerhovsky Z Soucek P. CYP2A13, ADH1B, and ADH1C Gene Polymorphisms and Pancreatic Cancer Risk. Pancreas (2010) 39(2):144–8. doi: 10.1097/MPA.0b013e3181bab6c2 19812523

[45] Brunner C Davies NM Martin RM Eeles R Easton D Kote-Jarai Z. Alcohol Consumption and Prostate Cancer Incidence and Progression: A Mendelian Randomisation Study. Int J Cancer (2017) 140(1):75–85. doi: 10.1002/ijc.30436 27643404 PMC5111609

[46] Michael J Howard LE Markt SC De Hoedt A Bailey C Mucci LA. Early-Life Alcohol Intake and High-Grade Prostate Cancer: Results From an Equal-Access, Racially Diverse Biopsy Cohort. Cancer Prev Res (Phila) (2018) 11(10):621–8. doi: 10.1158/1940-6207.CAPR-18-0057 30139875

[47] Hong S Khil H Lee DH Keum N Giovannucci EL. Alcohol Consumption and the Risk of Prostate Cancer: A Dose-Response Meta-Analysis. Nutrients (2020) 12(8):2188. doi: 10.3390/nu12082188 PMC7468718 32717903

[48] Zhao J Stockwell T Roemer A Chikritzhs T. Is Alcohol Consumption a Risk Factor for Prostate Cancer? A Systematic Review and Meta-Analysis. BMC Cancer (2016) 16(1):845. doi: 10.1186/s12885-016-2891-z 27842506 PMC5109713

[49] de Menezes RF Bergmann A Thuler LC. Alcohol Consumption and Risk of Cancer: A Systematic Literature Review. Asian Pac J Cancer Prev (2013) 14(9):4965–72. doi: 10.7314/APJCP.2013.14.9.4965 24175760

[50] Shimada K Anai S Fujii T Tanaka N Fujimoto K Konishi N. Syndecan-1 (CD138) Contributes to Prostate Cancer Progression by Stabilizing Tumour-Initiating Cells. J Pathol (2013) 231(4):495–504. doi: 10.1002/path.4271 24549646

[51] Santos NJ Barquilha CN Barbosa IC Macedo RT Lima FO Justulin LA. Syndecan Family Gene and Protein Expression and Their Prognostic Values for Prostate Cancer. Int J Mol Sci (2021) 22(16):8669. doi: 10.3390/ijms22168669 34445387 PMC8395474

[52] Szarvas T Reis H Vom Dorp F Tschirdewahn S Niedworok C Nyirady P. Soluble Syndecan-1 (SDC1) Serum Level as an Independent Pre-Operative Predictor of Cancer-Specific Survival in Prostate Cancer. Prostate (2016) 76(11):977–85. doi: 10.1002/pros.23186 27062540

[53] Szarvas T Sevcenco S Modos O Keresztes D Nyirády P Kubik A. Circulating Syndecan-1 Is Associated With Chemotherapy-Resistance in Castration-Resistant Prostate Cancer. Urol Oncol (2018) 36(6):312.e9–e15. doi: 10.1016/j.urolonc.2018.03.010 29628317

[54] Garner H de Visser KE. Immune Crosstalk in Cancer Progression and Metastatic Spread: A Complex Conversation. Nat Rev Immunol (2020) 20(8):483–97. doi: 10.1038/s41577-019-0271-z 32024984

[55] Hayashida K Parks WC Park PW. Syndecan-1 Shedding Facilitates the Resolution of Neutrophilic Inflammation by Removing Sequestered CXC Chemokines. Blood (2009) 114(14):3033–43. doi: 10.1182/blood-2009-02-204966 PMC2756208 19638625

[56] Laurent V Toulet A Attane C Milhas D Dauvillier S Zaidi F. Periprostatic Adipose Tissue Favors Prostate Cancer Cell Invasion in an Obesity-Dependent Manner: Role of Oxidative Stress. Mol Cancer Res (2019) 17(3):821–35. doi: 10.1158/1541-7786.MCR-18-0748 30606769

[57] Regős E Abdelfattah HH Reszegi A Szilák L Werling K Szabó G. Syndecan-1 Inhibits Early Stages of Liver Fibrogenesis by Interfering With Tgfβ1 Action and Upregulating MMP14. Matrix Biol (2018) 68-69:474–89. doi: 10.1016/j.matbio.2018.02.008 29454902

[58] Guan B Pungaliya P Li X Uquillas C Mutton LN Rubin EH. Ubiquitination by TOPORS Regulates the Prostate Tumor Suppressor NKX3.1. J Biol Chem (2008) 283(8):4834–40. doi: 10.1074/jbc.M708630200 18077445

[59] Johnson TR Khandrika L Kumar B Venezia S Koul S Chandhoke R. Focal Adhesion Kinase Controls Aggressive Phenotype of Androgen-Independent Prostate Cancer. Mol Cancer Res (2008) 6(10):1639–48. doi: 10.1158/1541-7786.MCR-08-0052 18922979

[60] Van Slambrouck S Jenkins AR Romero AE Steelant WF. Reorganization of the Integrin Alpha2 Subunit Controls Cell Adhesion and Cancer Cell Invasion in Prostate Cancer. Int J Oncol (2009) 34(6):1717–26. doi: 10.3892/ijo_00000302 PMC3235691 19424590

[61] Bianchi-Frias D Damodarasamy M Hernandez SA Gil da Costa RM Vakar-Lopez F Coleman IM. The Aged Microenvironment Influences the Tumorigenic Potential of Malignant Prostate Epithelial Cells. Mol Cancer Res (2019) 17(1):321–31. doi: 10.1158/1541-7786.MCR-18-0522 30224545

[62] Bellelli E Bracchi U Tanzi ML Benaglia G Montanarini G. Poliomyelitis Immunity Status at Different Intervals From Vaccination. Eur J Epidemiol (1986) 2(3):197–204. doi: 10.1007/BF00211532 3025013

[63] Graham TR Zhau HE Odero-Marah VA Osunkoya AO Kimbro KS Tighiouart M. Insulin-Like Growth Factor-I-Dependent Up-Regulation of ZEB1 Drives Epithelial-to-Mesenchymal Transition in Human Prostate Cancer Cells. Cancer Res (2008) 68(7):2479–88. doi: 10.1158/0008-5472.CAN-07-2559 18381457

[64] Gennigens C Menetrier-Caux C Droz JP. Insulin-Like Growth Factor (IGF) Family and Prostate Cancer. Crit Rev Oncol Hematol (2006) 58(2):124–45. doi: 10.1016/j.critrevonc.2005.10.003 16387509

[65] Odero-Marah V Hawsawi O Henderson V Sweeney J. Epithelial-Mesenchymal Transition (EMT) and Prostate Cancer. Adv Exp Med Biol (2018) 1095:101–10. doi: 10.1007/978-3-319-95693-0_6 30229551

[66] Sandsmark E Hansen AF Selnaes KM Bertilsson H Bofin AM Wright AJ. A Novel Non-Canonical Wnt Signature for Prostate Cancer Aggressiveness. Oncotarget (2017) 8(6):9572–86. doi: 10.18632/oncotarget.14161 PMC5354754 28030815

[67] Hu M Xie J Liu Z Wang X Liu M Wang J. Comprehensive Analysis Identifying Wnt Ligands Gene Family for Biochemical Recurrence in Prostate Adenocarcinoma and Construction of a Nomogram. J Comput Biol (2020) 27(12):1656–67. doi: 10.1089/cmb.2019.0397 32298604

[68] Gupta S Roy A Dwarakanath BS. Metabolic Cooperation and Competition in the Tumor Microenvironment: Implications for Therapy. Front Oncol (2017) 7:68. doi: 10.3389/fonc.2017.00068 28447025 PMC5388702

[69] Marchiq I Pouyssegur J. Hypoxia, Cancer Metabolism and the Therapeutic Benefit of Targeting Lactate/H(+) Symporters. J Mol Med (Berl) (2016) 94(2):155–71. doi: 10.1007/s00109-015-1307-x PMC4762928 26099350

[70] Mu X Shi W Xu Y Xu C Zhao T Geng B. Tumor-Derived Lactate Induces M2 Macrophage Polarization *via* the Activation of the ERK/STAT3 Signaling Pathway in Breast Cancer. Cell Cycle (2018) 17(4):428–38. doi: 10.1080/15384101.2018.1444305 PMC5927648 29468929

[71] Zhang L Li S. Lactic Acid Promotes Macrophage Polarization Through MCT-HIF1alpha Signaling in Gastric Cancer. Exp Cell Res (2020) 388(2):111846. doi: 10.1016/j.yexcr.2020.111846 31945319

[72] Fiaschi T Marini A Giannoni E Taddei ML Gandellini P De Donatis A. Reciprocal Metabolic Reprogramming Through Lactate Shuttle Coordinately Influences Tumor-Stroma Interplay. Cancer Res (2012) 72(19):5130–40. doi: 10.1158/0008-5472.CAN-12-1949 22850421

[73] Ippolito L Morandi A Taddei ML Parri M Comito G Iscaro A. Cancer-Associated Fibroblasts Promote Prostate Cancer Malignancy *via* Metabolic Rewiring and Mitochondrial Transfer. Oncogene (2019) 38(27):5339–55. doi: 10.1038/s41388-019-0805-7 30936458

[74] Keto CJ Aronson WJ Terris MK Presti JC Kane CJ Amling CL. Obesity Is Associated With Castration-Resistant Disease and Metastasis in Men Treated With Androgen Deprivation Therapy After Radical Prostatectomy: Results From the SEARCH Database. BJU Int (2012) 110(4):492–8. doi: 10.1111/j.1464-410X.2011.10754.x PMC3343219 22094083

[75] Muller TD Lee SJ Jastroch M Kabra D Stemmer K Aichler M. P62 Links Beta-Adrenergic Input to Mitochondrial Function and Thermogenesis. J Clin Invest (2013) 123(1):469–78. doi: 10.1172/JCI64209 PMC3533288 23257354

[76] Huang J Duran A Reina-Campos M Valencia T Castilla EA Müller TD. Adipocyte P62/SQSTM1 Suppresses Tumorigenesis Through Opposite Regulations of Metabolism in Adipose Tissue and Tumor. Cancer Cell (2018) 33(4):770–84.e6. doi: 10.1016/j.ccell.2018.03.001 29634950 PMC5896786

[77] Fong L Hotson A Powderly JD Sznol M Heist RS Choueiri TK. Adenosine 2a Receptor Blockade as an Immunotherapy for Treatment-Refractory Renal Cell Cancer. Cancer Discov (2020) 10(1):40–53. doi: 10.1158/2159-8290.CD-19-0980 31732494 PMC6954326

[78] Wang Q Ye Y Yu H Lin SH Tu H Liang D. Immune Checkpoint-Related Serum Proteins and Genetic Variants Predict Outcomes of Localized Prostate Cancer, a Cohort Study. Cancer Immunol Immunother (2021) 70(3):701–12. doi: 10.1007/s00262-020-02718-1 PMC7907032 32909077

[79] Tu TC Brown NK Kim TJ Wroblewska J Yang X Guo X. CD160 Is Essential for NK-Mediated IFN-Gamma Production. J Exp Med (2015) 212(3):415–29. doi: 10.1084/jem.20131601 PMC4354368 25711213

[80] Sun H Xu J Huang Q Huang M Li K Qu K. Reduced CD160 Expression Contributes to Impaired NK-Cell Function and Poor Clinical Outcomes in Patients With HCC. Cancer Res (2018) 78(23):6581–93. doi: 10.1158/0008-5472.CAN-18-1049 30232222

[81] Slebioda TJ Rowley TF Ferdinand JR Willoughby JE Buchan SL Taraban VY. Triggering of TNFRSF25 Promotes CD8(+) T-Cell Responses and Anti-Tumor Immunity. Eur J Immunol (2011) 41(9):2606–11. doi: 10.1002/eji.201141477 21688261

