# Supplementary material for: Energy Metabolism-Related Gene Prognostic Index Predicts Biochemical Recurrence for Patients With Prostate Cancer Undergoing Radical Prostatectomy
Source: Front Immunol. 2022 Feb 24;13:839362. doi: 10.3389/fimmu.2022.839362 (PMC8908254; doi:10.3389/fimmu.2022.839362)
Supplement: Supplementary Table 1 — The baselines of patients with prostate cancer from the TCGA database. BCR, biochemical recurrence; GS, Gleason score; IQR, interquartile range. [file Table_1.docx]

Supplementary table 1. The baselines of patients with prostate cancer from the TCGA database.

| Characteristic | No BCR | BCR | P value |
| --- | --- | --- | --- |
| Samples | 372 | 58 |  |
| Age, median (IQR) | 61 (56, 66) | 61 (57.5, 66) | 0.665 |
| N stage, n (%) |  |  | 0.083 |
| N0 | 267 (87.3%) | 39 (12.7%) |  |
| N1 | 54 (78.3%) | 15 (21.7%) |  |
| Positive lymphnodes, n (%) |  |  | 0.057 |
| No | 254 (88.2%) | 34 (11.8%) |  |
| Yes | 55 (78.6%) | 15 (21.4%) |  |
| Residual tumor, n (%) |  |  | 0.165 |
| No | 241 (88.3%) | 32 (11.7%) |  |
| Yes | 121 (82.9%) | 25 (17.1%) |  |
| Gleason score, n (%) |  |  | < 0.001 |
| GS=6 | 37 (94.9%) | 2 (5.1%) |  |
| GS=7 | 193 (93.7%) | 13 (6.3%) |  |
| GS=8 | 48 (81.4%) | 11 (18.6%) |  |
| GS=9 | 94 (74.6%) | 32 (25.4%) |  |
| T stage, n (%) |  |  | < 0.001 |
| T2 | 149 (96.1%) | 6 (3.9%) |  |
| T3-4 | 218 (81%) | 51 (19%) |  |
| Race, n (%) |  |  | 0.831 |
| ASIAN | 9 (81.8%) | 2 (18.2%) |  |
| BLACK OR AFRICAN AMERICAN | 43 (86%) | 7 (14%) |  |
| WHITE | 307 (86.5%) | 48 (13.5%) |  |

BCR: biochemical recurrence; GS: Gleason score; IQR: interquartile range.

Supplementary table 2. Univariate and multivariate COX regression analysis of EMGPI and clinical indicators for the paitents with prostate cancer from the TCGA database.

| Characteristics | Total(N) | Univariate analysis | |  | Multivariate analysis | |
| --- | --- | --- | --- | --- | --- | --- |
|  |  | Hazard ratio (95% CI) | P value |  | Hazard ratio (95% CI) | P value |
| EMRGPI | 430 |  |  |  |  |  |
| Low | 215 | Reference |  |  |  |  |
| High | 215 | 2.020 (1.171-3.486) | **0.012** |  | 1.965 (1.056-3.656) | **0.033** |
| Age | 430 | 1.016 (0.978-1.055) | 0.426 |  |  |  |
| N stage | 375 |  |  |  |  |  |
| N0 | 306 | Reference |  |  |  |  |
| N1 | 69 | 1.822 (1.001-3.313) | **0.049** |  | 78074549.080 (0.000-Inf) | 0.999 |
| Positive lymphnodes | 358 |  |  |  |  |  |
| No | 288 | Reference |  |  |  |  |
| Yes | 70 | 1.937 (1.051-3.567) | **0.034** |  | 0.000 (0.000-Inf) | 0.999 |
| Residual tumor | 419 |  |  |  |  |  |
| No | 273 | Reference |  |  |  |  |
| Yes | 146 | 1.781 (1.050-3.019) | **0.032** |  | 1.025 (0.548-1.919) | 0.938 |
| Gleason score | 430 |  |  |  |  |  |
| GS=6 | 39 | Reference |  |  |  |  |
| GS=9 | 126 | 4.833 (1.157-20.194) | **0.031** |  | 23259655.899 (0.000-Inf) | 0.996 |
| GS=8 | 59 | 3.763 (0.832-17.011) | 0.085 |  | 24378969.744 (0.000-Inf) | 0.996 |
| GS=7 | 206 | 1.072 (0.242-4.756) | 0.927 |  | 7961981.198 (0.000-Inf) | 0.996 |
| T stage | 424 |  |  |  |  |  |
| T2 | 155 | Reference |  |  |  |  |
| T3-4 | 269 | 5.238 (2.247-12.213) | **<0.001** |  | 3.193 (1.093-9.328) | **0.034** |
| Race | 416 |  |  |  |  |  |
| WHITE | 355 | Reference |  |  |  |  |
| ASIAN | 11 | 0.673 (0.147-3.079) | 0.610 |  |  |  |
| BLACK OR AFRICAN AMERICAN | 50 | 0.648 (0.288-1.460) | 0.296 |  |  |  |

EMRGPI: energy metabolism-related gene prognositc index; GS: Gleason score.
